# Beyond the Swelling: A Missed Foreign Body in the Testis

**DOI:** 10.7759/cureus.91717

**Published:** 2025-09-06

**Authors:** Jan Stępka, Tomasz Milecki, Andrzej Bałoniak, Konstanty Drogowski, Wojciech Cieślikowski, Tomasz Deja

**Affiliations:** 1 Department of Urology, Ministry of Internal Affairs Hospital, Poznań, POL; 2 Department of Urology and Oncological Urology, University Clinical Hospital, Poznań, POL; 3 Department of Urology, Poznań University of Medical Sciences, Poznań, POL

**Keywords:** acoustic shadowing, asymptomatic presentation, delayed diagnosis, penetrating injury, scrotal trauma, soft tissue foreign body, surgical removal, testicular foreign body, ultrasonography, urology case report

## Abstract

Intratesticular foreign bodies are rare. We present the case of a young man who, three months after experiencing scrotal trauma, was evaluated for a suspected intratesticular foreign body. The incident occurred during the use of a wire brush attached to a grinder, likely resulting in the penetration of a small metallic fragment into the scrotal wall and testicular tissue. Initial ultrasound imaging was inconclusive, and the patient was treated empirically with antibiotics. Although his symptoms resolved over time, ongoing concern led to multiple follow-up visits. A third ultrasound performed approximately 12 weeks post-injury revealed a linear, hyperechoic structure, raising suspicion for a retained foreign body and prompting surgical referral. Upon admission, the patient was asymptomatic, and physical examination revealed only a small, firm nodule in the left testis. Surgical exploration confirmed the presence of a thin metallic wire, which was removed without complications. This case illustrates the diagnostic difficulty of identifying retained intratesticular foreign bodies when early imaging is non-diagnostic and symptoms subside. It emphasizes the value of repeated high-resolution ultrasonography in cases with a suggestive clinical history.

## Introduction

Scrotal trauma is rare, as it comprises less than 1% of all trauma presentations in emergency departments in the United States [1]. Among these injuries, blunt trauma is the most common as it accounts for about 80% of all scrotal injuries, usually due to car accidents, sports injuries or falls [2,3]. Penetrating injuries are much less frequent. In a retrospective analysis by Tkocz and Kupajski penetrating scrotal trauma accounted for only about 10% of all testicular injuries over a four-year period in a single-center experience [4].

Penetrating testicular trauma may result in retention of a foreign body within the scrotal sac or testicular parenchyma. Such cases are rare and typically result from self-inflicted injuries, construction site injuries, or intentional insertion [5-8]. Diagnosis can be challenging, especially with symptoms of swelling and may be delayed due to non-specific clinical signs or inconclusive imaging results [9].

In most cases, retained foreign bodies in the testis following penetrating trauma present with persistent symptoms such as pain, swelling, or infection [7-9]. However, to our knowledge, no previous reports describe a case in which a penetrating scrotal injury resulted in intratesticular foreign body retention with spontaneously resolving acute symptoms, ultimately leaving the patient asymptomatic over time. Here we present a case of asymptomatic, retained metallic foreign body in the testis, diagnosed several weeks after penetrating scrotal trauma and managed with surgical excision. This case highlights the diagnostic difficulty of subtle or delayed imaging findings and the importance of correlating clinical history with repeated high-resolution ultrasonography in suspected penetrating scrotal injuries.

## Case presentation

**Figure 1:**
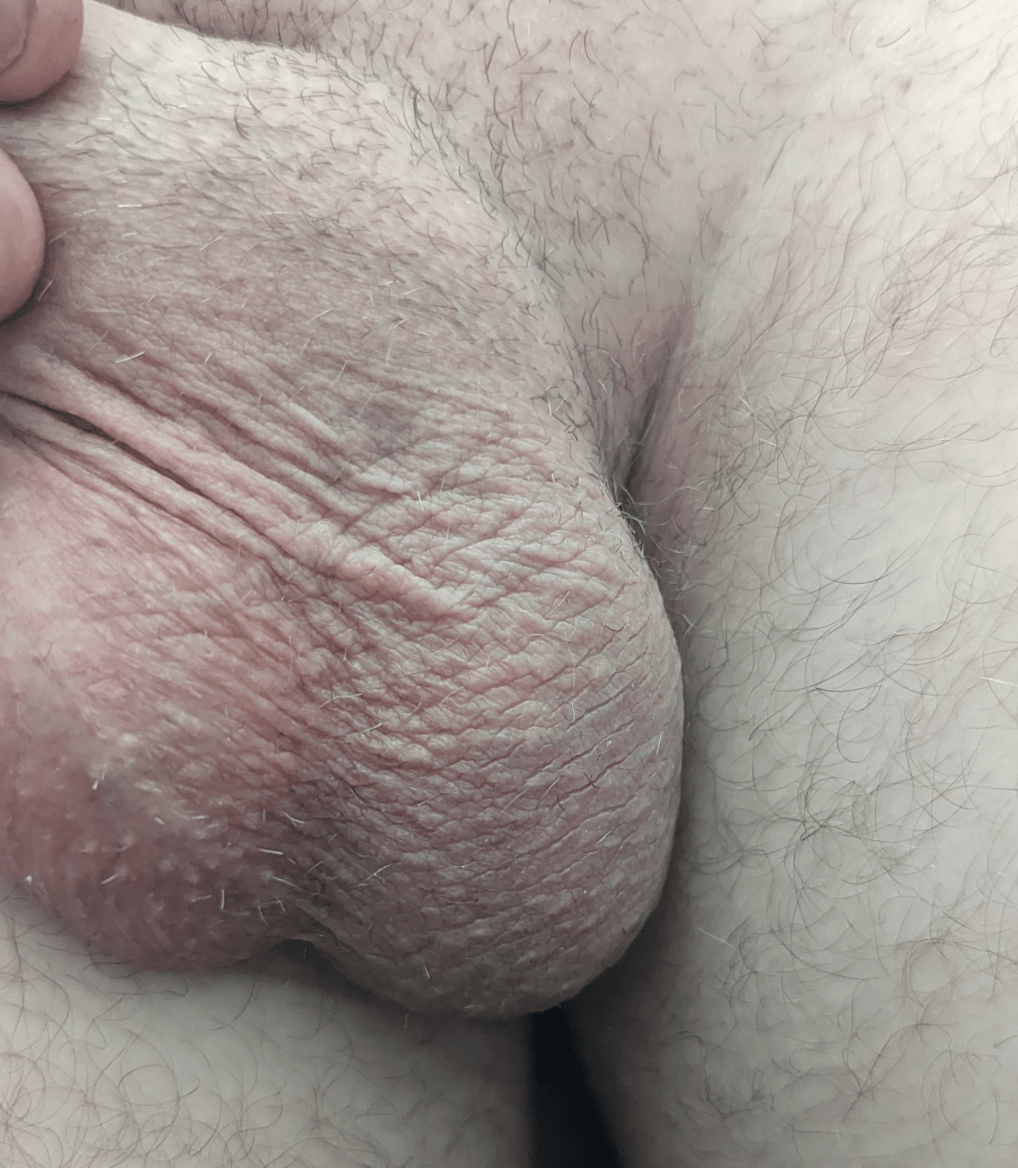
External appearance of the scrotum on admission. The scrotum appears normal in size, shape, and symmetry, with no signs of erythema, edema, or skin lesions. This clinical image illustrates the absence of visible abnormalities despite the confirmed presence of an intratesticular foreign body.

A young, 32-year-old, professionally active male patient was referred to the urology department with a three-month history of suspected foreign body in the left testis following a work-related injury. The trauma occurred while using a wire brush mounted on a grinder, during which a small metal fragment likely penetrated the scrotum and embedded within the testicular parenchyma. In the acute phase, the patient experienced significant pain and noticeable scrotal swelling, prompting a private urological consultation. As reported by the patient, during the initial urological visit at another facility, the ultrasound did not reveal any foreign body. He was treated empirically with ciprofloxacin and symptomatic management. Over the following weeks, the symptoms gradually subsided, but the patient remained concerned due to palpable firm nodule in the testis. He therefore sought another urological consultation at a different facility, where, according to the patient's account, no foreign body was detected and analgesics were prescribed. Approximately 11.5 weeks post-injury, still concerned, the patient decided to seek further consultation - this time with a physician from our department. A third ultrasound finally revealed a suspicious hyperechoic linear structure in the left testis, raising concern for a retained foreign body. The patient was then referred for inpatient management. Upon admission, he was asymptomatic and in good general condition. The scrotum appeared entirely normal - symmetrical, without erythema, pain, swelling, or skin lesions - as seen in Figure 1. On palpation, a small, firm nodule was detectable within the left testis.

**Figure 2:**
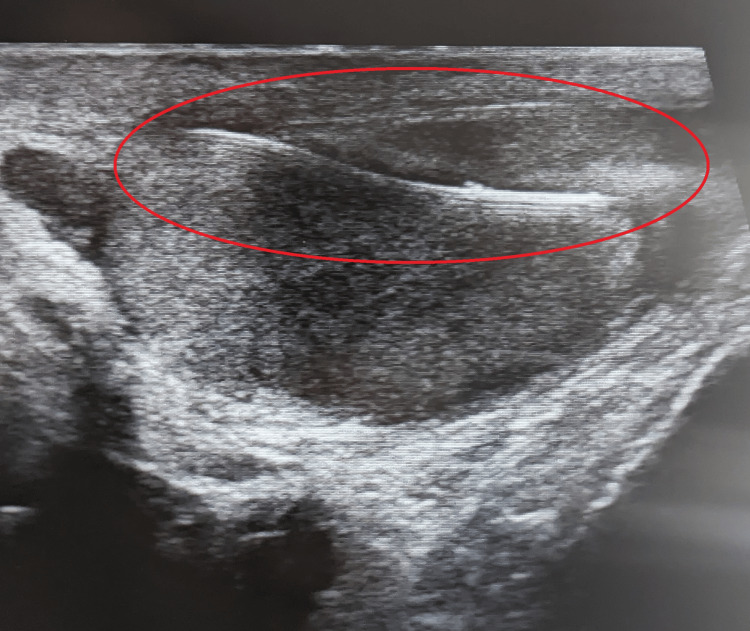
Ultrasonographic visualization of a retained metallic foreign body in the left testis. High-resolution ultrasound demonstrates a linear, hyperechoic structure traversing the testicular parenchyma (red circle). These sonographic features are characteristic of metallic objects and confirm the intratesticular location of the foreign body. Probe alignment and angle were adjusted to optimize visibility.

Repeat ultrasonography confirmed the presence of a hyperechoic, linear structure with distinct posterior acoustic shadowing traversing the testicular parenchyma (as demonstrated in Figure 2 and Video 1), consistent with a metallic foreign body. The patient also reported sporadically triggering airport metal detectors, supporting the presumed metallic composition of the object.

**Video 1:** Dynamic ultrasound recording of the retained metallic foreign body. Ultrasound clip showing the echogenic, linear structure within the left testis, consistent with a metallic foreign body.

**Figure 3:**
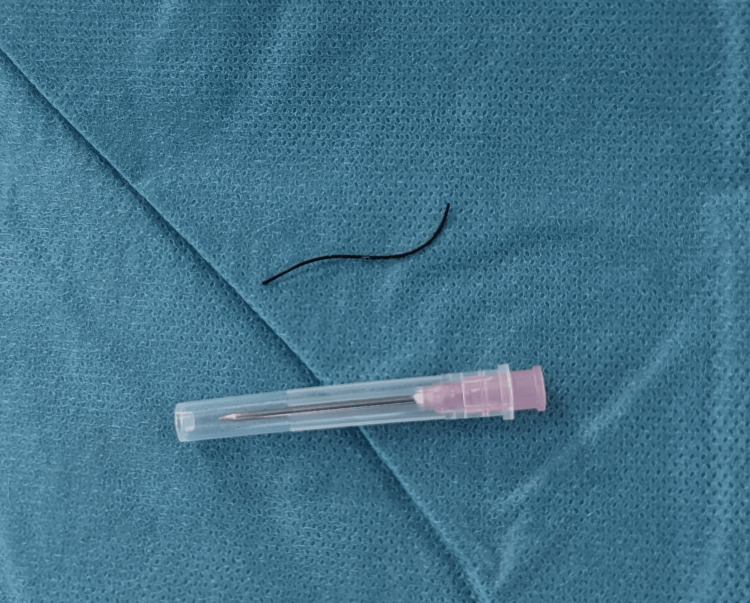
Extracted intratesticular foreign body A thin, sharp-ended metallic wire removed from the left testis, consistent with the patient’s reported mechanism of injury. The pink intravenous needle placed adjacent provides size reference. This image confirms the presence and physical characteristics of the foreign object visualized in prior imaging.

Under general anesthesia, a transverse left scrotal incision was made. The testis was carefully dissected, and a thin, thread-like metallic wire was visualized protruding slightly from the lateral surface. The foreign body was extracted in one piece (Figure 3). Hemostasis was achieved, and the wound was closed in layers. The patient’s postoperative course was uneventful. He was discharged two days later and reported no complaints at the follow-up visit after two months.

## Discussion

This case highlights the diagnostic challenge posed by retained foreign bodies in the testis, particularly when initial imaging is negative and symptoms are minimal or absent. In the early post-traumatic phase, tissue edema and inflammation may obscure sonographic detail, reducing sensitivity [9,10]. Moreover, the orientation of the metallic object relative to the ultrasound beam can hinder visualization, especially when aligned parallel to the beam path [11]. Ultrasound remains the primary imaging modality for detecting soft tissue and intratesticular foreign bodies. According to Davis et al., ultrasound has a sensitivity of 72% and a specificity up to 92% for detecting foreign materials [12]. However, it is worth mentioning that, when ultrasound results are negative and clinical suspicion remains, a non-contrast CT scan is preferred, especially for detecting objects such as metal or glass, which are clearly visible on CT [13]. In this case, the object appeared as a sharply defined echogenic line with posterior acoustic shadowing, a classic sonographic signature of metallic structures - as clearly seen in Figure 2 and Video 1. It is worth noting that visualizing the object in this manner was not easy and required considerable manipulation of the probe. When the probe was applied without adjustment, the object appeared only as a small, hyperechoic dot. These findings illustrate the diagnostic value of repeating high-resolution ultrasound scans when initial evaluations are inconclusive. Long-term intratesticular retention of a foreign body may lead to serious complications, including granuloma formation, necrosis, chronic infection, and impaired fertility [14,15]. These risks justify surgical intervention even in asymptomatic patients, particularly when the object is metallic and potentially reactive. Differential diagnosis should include microcalcifications, epidermoid cysts, or ultrasonographic artifacts [10,14]. However, clinical history of penetrating trauma, physical findings, and characteristic imaging help confirm the diagnosis. This case also serves as a reminder that normal scrotal appearance (Figure 1) does not exclude the presence of penetrating injury.

## Conclusions

This case highlights the fact that small intratesticular foreign bodies may go undetected for weeks due to swelling and other acute-phase symptoms. When the mechanism of injury raises suspicion of a retained object, repeated imaging may prove valuable - even if the patient is asymptomatic. Early removal of the foreign body is important, as it reduces the risk of complications such as infection, granuloma formation, or testicular damage. Recognizing subtle clinical signs can play a crucial role in maintaining testicular health.

## References

[1] Grigorian A, Livingston JK, Schubl SD (2018). National analysis of testicular and scrotal trauma in the USA. Res Rep Urol.

[2] (2025). EAU Guidelines on Urological Trauma. https://d56bochluxqnz.cloudfront.net/documents/full-guideline/EAU-Guidelines-on-Urological-Trauma-2025.pdf.

[3] Hunter SR, Lishnak TS, Powers AM, Lisle DK (2013). Male genital trauma in sports. Clin Sports Med.

[4] Tkocz M, Kupajski M (2008). The injures of the male external genital organs in the own material. Chirurgia Polska (Polish Surgery).

[5] Randhawa H, Blankstein U, Davies T (2019). Scrotal trauma: a case report and review of the literature. Can Urol Assoc J.

[6] Trigui M, Ouanes Y, Chaker K, Marrak M, Madani MA, Nouira Y (2023). Intrascrotal self insertion of foreign body: form of entry to schizophrenia. Urol Case Rep.

[7] Migliorini F, Bizzotto L, Curti P, Porcaro AB, Artibani W (2017). An unusual case of pneumatic nail gun scrotal injury and revision of the literature. Arch Ital Urol Androl.

[8] Pavia MP, Fabiani A, Principi E, Servi L (2021). Ultrasound of a patient with penetrating scrotal trauma: finding a needle in a haystack. Radiol Case Rep.

[9] Mante SD, Yeboah ED, Adusei B, Edusa S (2013). Foreign body in scrotum following a boat engine blast accident. Ghana Med J.

[10] Bhatt S, Dogra VS (2008). Role of US in testicular and scrotal trauma. Radiographics.

[11] Farahmand S, Bagheri-Hariri S, Mehran S, Arbab M, Khazaeipour Z, Basir-Ghafouri H, Saeedi M (2014). A simplified training method for soft tissue foreign body detection using ultrasound in emergency medicine residency program. Ultrasound.

[12] Davis J, Czerniski B, Au A, Adhikari S, Farrell I, Fields JM (2015). Diagnostic accuracy of ultrasonography in retained soft tissue foreign bodies: a systematic review and meta-analysis. Acad Emerg Med.

[13] Maralakunte M, Debi U, Singh L, Pruthi H, Bhatia V, Devi G, MS S (2020). Foreign body imaging-experience with 6 cases of retained foreign bodies in the emergency radiology unit. Arch Clin Med Case Rep.

[14] Kim W, Rosen MA, Langer JE, Banner MP, Siegelman ES, Ramchandani P (2007). US MR imaging correlation in pathologic conditions of the scrotum. Radiographics.

[15] Fijak M, Pilatz A, Hedger MP (2018). Infectious, inflammatory and ‘autoimmune’ male factor infertility: how do rodent models inform clinical practice?. Hum Reprod Update.

